# IL-21 mediates microRNA-423-5p /claudin-5 signal pathway and intestinal barrier function in inflammatory bowel disease

**DOI:** 10.18632/aging.103566

**Published:** 2020-08-28

**Authors:** Mu Wang, Jian Guo, Yi-Qing Zhao, Jun-Ping Wang

**Affiliations:** 1 Department of Neurology, Shanxi Provincial People's Hospital, The Affiliated People's Hospital of Shanxi Medical University, Taiyuan 030012, Shanxi Province, China; 2 Department of Metabolism, Digestion and Reproduction, Faculty of Medicine, Imperial College London, London W12 0NN, United Kingdom; 3 The Institutes of Biomedical Sciences, Shanxi University, Taiyuan 030006, Shanxi Province, China; 4 Department of General Surgery, Shanxi Provincial People's Hospital, The Affiliated People's Hospital of Shanxi Medical University, Taiyuan 030012, Shanxi Province, China; 5 Department of Gastroenterology, Shanxi Provincial People's Hospital, The Affiliated People's Hospital of Shanxi Medical University, Taiyuan 030001, Shanxi Province, China

**Keywords:** IBD, inflammation, microRNA-423-5p, IL-21, claudin-5

## Abstract

Inflammatory bowel disease (IBD) is a group of chronic and recurrent nonspecific inflammatory disorders, including Crohn's disease (CD) and ulcerative colitis (UC). Due to the persistent inflammation of intestinal mucosa caused by immune disorders, barrier dysfunction may be an essential cause of the pathogenesis of IBD. Therefore, exploring the mechanism is very important to clarify the pathogenesis of IBD. In our research, we provided evidence of IL-21 function in IBD. The junction complex protein claudin-5 may be a downstream gene of the IL-21. Anti-IL-21 administrated prevented DSS-simulative colitis via recovering claudin-5 expression in the human colonic epithelial cells. Meanwhile, we described that miR-423-5p could be involved in IL-21/ claudin-5 pathway by regulating NF-κB/MAPKs/JNK signaling pathway, which may provide a new therapeutic target for IBD.

## INTRODUCTION

IBD is a chronic nonspecific inflammatory disease of the intestinal tract, with diarrhea, abdominal pain, and bloody stool as its primary clinical manifestations, which is a common autoimmune disease in China and many European countries and America in recent years [1, 2]. It is mainly caused by intestinal barrier dysfunction. At present, the pathology and etiological mechanism of IBD still need further exploration.

Interleukin-21 (IL-21) is mainly derived from one of the living CD4T cells shared with other members (IL-2, IL-4, IL-13, and IL-15) and the γc receptor subunit of helper T cells (Th). IL-21 receptor (IL-21R) contains IL-21Rα and γc subunit. IL-21 is an essential member of the IL-2 family. IL-21 is expressed in thymus, spleen, and other lymphoid tissues. After binding to IL-21R, IL-21 promotes the activation of transcription factors such as STAT1 and STAT3 through the JAK/STAT signal pathway. IL-21 could regulate the proliferation of B cells, promote the production of immunoglobulin (Ig) G, and inhibit the production of IgE [3]. Previous studies have confirmed that in the process of inflammatory bowel disease, parasite infection, and other diseases, IL-21 stimulated T cell proliferation, promoted Th1 cell differentiation, increased the production of interferon (IFN)-γ, and aggravated the progression of inflammation. IL-21 can also improve the proliferation of CD8+ cytotoxic T lymphocytes and the creation of IFN-γ [4, 5]. IL-21 plays a vital role in the progression of inflammatory bowel disease caused, but its mechanism is still not very clear.

The intestinal barrier system is not only the first line of defense against human invasion of pathogens but also a prerequisite for maintaining the stability of the intestinal ecosystem. In a broad sense, the normal intestinal barrier is composed of the epithelial barrier, immune barrier, mucus barrier, and microecological barrier. The disorder of the intestinal barrier function can lead to acute diarrhea, inflammatory bowel disease recurrence, and other diseases [6]. The claudin protein family is a cytoskeleton protein with tight junctions between cells. It was first extracted from the chicken liver by Furuse et al. in 1998 [7], and the claudin-1 gene was cloned for the first time in 1999 [8]. Since then, other members of the claudin family have been gradually discovered. So far, 27 members of the claudin family have been cloned [9]. Claudin-5, a member of the Claudin family, also has the function of fence and barrier, that is, it can control the communication of ions between cells, prevent the mixing of different molecules in the apical membrane and basolateral membrane of epithelial cells, and play an essential role in maintaining cell polarity [10].

In our study, we revealed the potential mechanism of the IL-21 pathway in IBD. We confirmed that claudin-5 may be downstream of IL-21 and found that miR-423-5p played a crucial mediating function in regulating IL-21/claudin-5 pathway and intestinal barrier function by NF-κB/MAPKs/JNK signaling pathway. This newly discovered IL-21-miR-423-5p-claudin-5 pathway may be a target for IBD therapy.

## RESULTS

### Abnormally expression of IL-21 in ulcerative colitis patients

**Figure 1:**
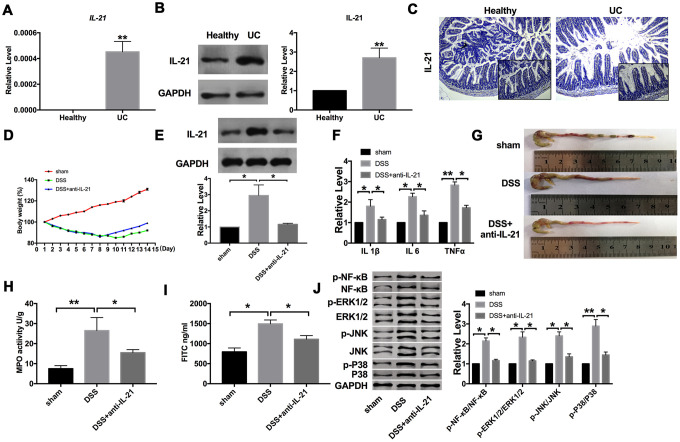
**Increased expression of IL-21 in UC patients**. (**A**) The expression of IL-21 in colonic mucosa from UC patients and healthy volunteers. n=15. (**B**) The protein level of IL-21in human samples. n=5. (**C**) Representative images of immunohistochemistry staining with IL-21. (**D**) Changes in body weight in different groups of mice. n=10. (**E**) The protein level of IL-21 in different groups. n=10. (**F**) The mRNA level of IL1β, IL6, and TNFα in plasma. n=5. (**G**) The length of the colon in different groups of mice. (**H**) MPO activity detection in different groups of mice.n=5. (**I**) Serum FITC- dextran was used as a monitoring index of intestinal permeability. n=6. (**J**) The protein level of NF-κB/MAPKs/JNK signaling pathway components in different groups. n=5 *P < 0.05, **P < 0.01**.**

IL-21 stimulates fibroblasts to secrete extracellular matrix-degrading enzymes and epithelial cells to secrete T cell chemical inducer MIP-3α, which is an inflammatory factor. To explore the role of IL-21 in IBD, we examined the expression of IL-21 in colonic mucosa from UC patients and healthy volunteers. RT-PCR results performed the significantly increased expression level of IL-21 in UC patients, while there was almost no expression of IL-21 in the normal colon tissues (Figure 1A). Meanwhile, the protein level of IL-21 was also upregulated in colonic mucosa from UC patients (Figure 1B). Similar results were also performed in the IHC staining of colonic samples (Figure 1C).

For further research, we established a C57BL/6 mouse model of acute colitis with DSS and treated it with IL-21 neutralizing antibody. Compared with the DSS group, the bodyweight of mice treated with anti-IL-21 recovered (Figure 1D). After assessing the level of IL-21, we found that DSS induced the expression of IL-21, which was recovered by anti-IL-21 (Figure 1E). Further, we found that DSS induced the expression of inflammatory cytokines (IL1β, IL6, and TNFα). Compared with the DSS group, the expression of IL1β, IL6, and TNFα were decreased in anti-IL-21 treated mice (Figure 1F). We found that mice treated with IL-21 neutralizing antibody had a protective effect on experimental colitis compared with the DSS group (Figure 1G). As a biochemical test of acute enteritis, colonic MPO activity also showed that IL-21 neutralizing antibody could significantly reduce inflammation (Figure 1H). Further, we detected the intestinal barrier function of mice, and the results showed that IL-21 neutralizing antibody could decrease the level of FD40 in the serum of DSS mice (Figure 1I). Meanwhile, we detected the components of the NF-κB/MAPKs/JNK signaling pathway. DSS-induced the phosphorylation of NF-κB, ERK1/2, JNK, and P38, which were inhibited by anti-IL-21 (Figure 1J). These results suggested that fighting against IL-21 can relieve the symptoms of UC.

### IL-21 regulates the intestinal epithelial barrier function by targeting claudin-5

**Figure 2:**
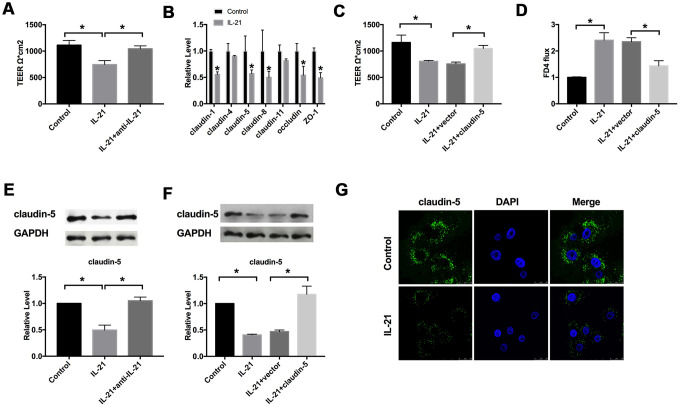
**IL-21 regulates the intestinal epithelial TJ barrier function by targeting claudin-5**. (**A**) The effect of IL-21 on intestinal epithelial barrier function. n=3. (**B**) RT-PCR was employed to detect the mRNA level of claudin-1, claudin-4, claudin-5, claudin-8, claudin-11, occludin, and ZO-1. n=7. (**C**) The effect of claudin-5 on TEER in Caco-2 cells. n=3. (**D**) The FD4 level was detected after claudin-5/vector transfection in IL-21 treated cells. n=5. (**E**, **F**) The protein level of claudin-5 in different groups. n=4. (**G**) Representative images of immunofluorescence staining with claudin-5. *P < 0.05, **P < 0.01.

The value of trans-epithelial resistance is an index to detect the tightness of colonic intercellular connection. The effect of IL-21 on intestinal epithelial barrier function was detected via TEER experiments. We found that IL-21 decreased the TEER, while IL-21 neutralizing antibodies markedly recovery the tight junction (Figure 2A).

The damage of the colonic mucosal barrier function is one of the pathogenesis of UC. Occludin, claudin, and zonulae occludente (ZO) in colonic epithelial cells play an essential role in intestinal tight junction function. We detected the level of claudin-1, claudin-4, claudin-5, claudin-8, claudin-11, occludin, and ZO-1 by employing the RT-PCR assay. We observed that claudin-1, claudin-5, claudin-8, occludin, and ZO-1 were decreased after IL-21 treatment in cells (Figure 2B). Except for claudin-1 and claudin-8, claudin-5 is the most significantly abnormal expression in Caco-2 cells after IL-21 treatment. We speculated that claudin-5 might be downstream of IL-21 in UC. Then we constructed the plasmid for upregulating the level of claudin-5 (claudin-5), vector plasmid (vector) was described as a negative control, we transfected claudin-5/vector into Caco-2 cells after treated with or without IL-21, we observed that overexpression of claudin-5 restored the injured-intestinal epithelial barrier function after IL-21 treatment (Figure 2C). We also evaluated the permeability of Caco-2 monolayer with fluorescein isothiocyanate-dexamethasone (FD4). Similar to the results of TEER, the overexpression of claudin-5 restored the change of FD 4 level caused by IL-21 (Figure 2D). Meanwhile, we assessed the protein level of claudin-5 in Caco-2 cells after IL-21 and IL-21 neutralizing antibodies treatment. Western blot assay results showed that IL-21 inhibited the level of claudin-5, and IL-21 neutralizing antibodies recovered the level of claudin-5 (Figure 2E, 2F). Further, we detected the expression of claudin-5 after IL-21 administrated by immunofluorescence experiment, similar to western blot assay, IL-21 blocked the expression of claudin-5 (Figure 2G). In summary, IL-21 regulated the function of the intestinal barrier by controlling the level of claudin-5.

### MiR-423-5p binds with 3’UTR of *claudin-5.*

**Figure 3:**
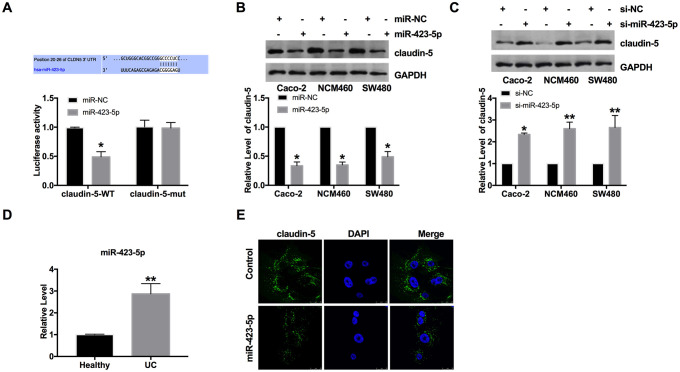
**MiR-423-5p could bind with the 3'UTR of *claudin-5.*** (**A**) Binding sequences between miR-423-5p and claudin-5 (CLDN5) (http://www.targetscan.org/cgi-bin/targetscan/vert_72/view_gene.cgi?rs=ENST00000406028.1&taxid=9606&members=miR-423-5p&showcnc=1&shownc=1&shownc_nc=1&showncf1=&showncf2=&subset=1). The luciferase assay report. n=3. (**B**, **C**) The expression of claudin-5 in colorectal epithelial cell lines. n=4. (**D**) The level of miR-423-5p in colonic mucosa from UC patients and healthy volunteers. n=15. (**E**) Representative images of immunofluorescence staining with claudin-5. *P < 0.05, **P < 0.01

Through the prediction of the bioinformatics website, we found that there are binding sequences between miR-423-5p and claudin-5. By performing luciferase assay, we ensured that miR-423-5p could interact and bind with 3'UTR of *claudin-5* (Figure 3A). Then we transfected the miR-423-5p mimic/miR-NC into three colorectal epithelial cells (Caco-2, NCM460, and SW480 cells). Then the protein level of claudin-5 was assessed in different cell lines. Figure 3B showed that forced expression of miR-423-5p could inhibit the level of claudin-5. Further, si-miR-423-5p/si-NC were transfected into cells for detecting the level of claudin-5. The assays performed, blocking the level of miR-423-5p would restore the level of claudin-5 (Figure 3C). Meanwhile, we found that the level of miR-423-5p was abnormally elevated in UC patients (Figure 3D). Further, we detected the level of claudin-5 in Caco-2 cells by immunofluorescence experiment, similar to western blot assay. MiR-423-5p blocked the expression of claudin-5 (Figure 3E).

### MiR-423-5p regulates the intestinal epithelial barrier function by targeting claudin-5

**Figure 4:**
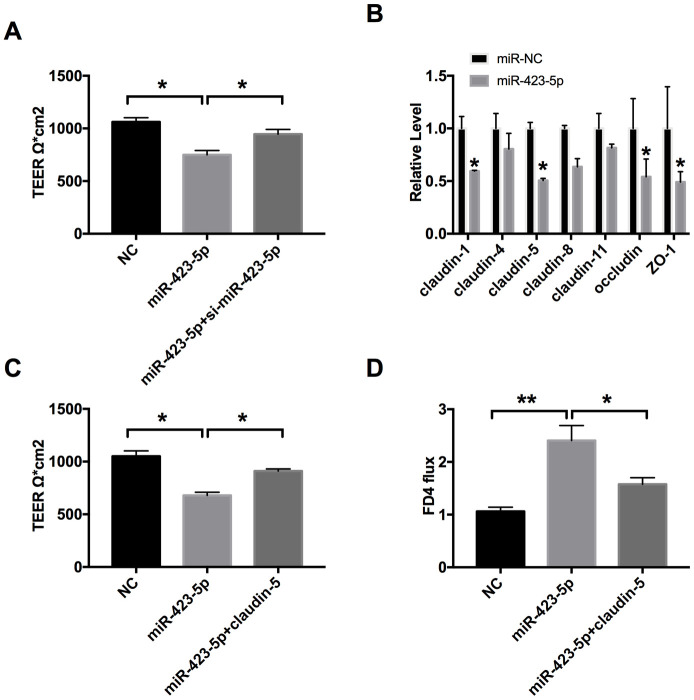
**MiR-423-5p regulates the intestinal epithelial barrier function by targeting claudin-5.** (**A**) The effect of miR-423-5p on intestinal epithelial barrier function. n=3. (**B**) RT-PCR was employed to detect the mRNA level of claudin-1, claudin-4, claudin-5, claudin-8, claudin-11, occludin, and ZO-1. n=6. (**C**) The effect of miR-423-5p and claudin-5 on TEER in Caco-2 cells. n=4. (**D**) The FD4 level was detected after claudin-5/vector transfection with miR-423-5p mimic/miR-NC. n=5. *P < 0.05, **P < 0.01.

We found that miR-423-5p mimic transfection reduced the TEER of Caco-2 cells. However, si-miR-423-5p significantly enhanced the tight junction of Caco-2 cells (Figure 4A). Then we detected the level of claudin-1, claudin-4, claudin-5, claudin-8, claudin-11, occludin, and ZO-1. We observed that the mRNA level of claudin-5 was also decreased after miR-423-5p mimic transfection in cells (Figure 4B). We transfected claudin-5 into Caco-2 cells after transfecting with miR-423-5p mimic/miR-NC, we observed that overexpression of claudin-5 restored the injured-intestinal epithelial barrier function after forced expression of miR-423-5p (Figure 4C). Similar to the results of TEER, the overexpression of claudin-5 restored the change of FD4 level caused by overexpression miR-423-5p (Figure 4D). Taken together, miR-423-5p would control the intestinal epithelial barrier function by targeting claudin-5.

### MiR-423-5p antagomir administrated performs protection in DSS mice.

**Figure 5:**
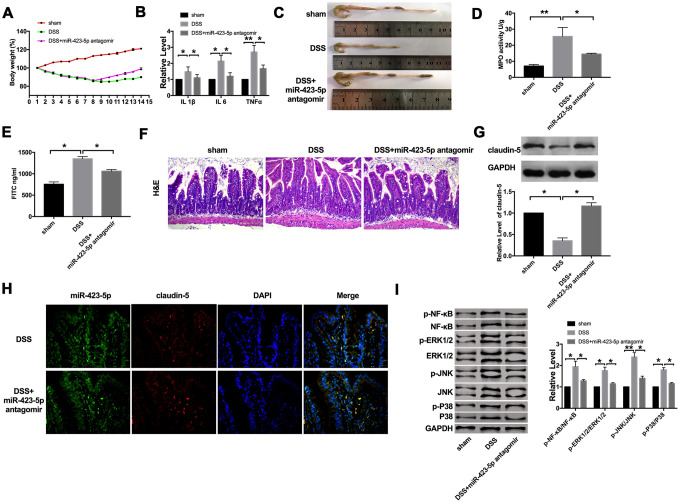
**The effect of miR-423-5p in DSS mice.** (**A**) Changes in body weight in different groups of mice. n=10. (**B**) The mRNA level of IL1β, IL6, and TNFα in plasma. n=5. (**C**) The length of the colon in different groups of mice. (**D**) MPO activity detection in different groups of mice. n=4. (**E**) Serum FITC- dextran was used as a monitoring index of intestinal permeability. n=5. (**F**) Representative images of H&E staining. (**G**) The expression of claudin-5 in different groups. n=4. (**H**) Representative images of immunofluorescence staining with claudin-5. (**I**) The protein level of NF-κB/MAPKs/JNK signaling pathway components in different groups. n=5. *P < 0.05, **P < 0.01.

Next, we established a C57BL/6 mouse model of acute colitis with DSS and treated it with miR-423-5p antagomir. Compared with the DSS group, the bodyweight of mice treated with miR-423-5p antagomir recovered (Figure 5A). Further, we found that DSS induced the expression of IL1β, IL6, and TNFα. Compared with the DSS group, the expression of IL1β, IL6 and TNFα were decreased in miR-423-5p antagomir injected mice (Figure 5B)

We also found that mice treated with miR-423-5p antagomir had a protective effect on experimental colitis compared with the DSS group (Figure 5C). Colonic MPO activity also showed that miR-423-5p antagomir could significantly reduce inflammation (Figure 5D). Further, miR-423-5p antagomir down-regulated the level of FD40 in the serum of DSS mice (Figure 5E). H&E staining also performed that miR-423-5p antagomir alleviated the inflammation level induced by DSS (Figure 5F). Meanwhile, we determined the level of claudin-5. MiR-423-5p antagomir restored the down-regulated of claudin-5 in DSS mice (Figure 5G). Consistent with the results of western blot, the immunofluorescence experiment was performed on rectal tissue sections, and it was found that the level of claudin-5 increased in DSS mice injected with miR-423-5p antagomir (Figure 5H). Meanwhile, we detected the components of the NF-κB/MAPKs/JNK signaling pathway. DSS-induced the phosphorylation of NF-κB, ERK1/2, JNK, and P38, which were inhibited by miR-423-5p antagomir (Figure 5I).

### Relationship between IL-21 and MiR-423-5p in UC

**Figure 6:**
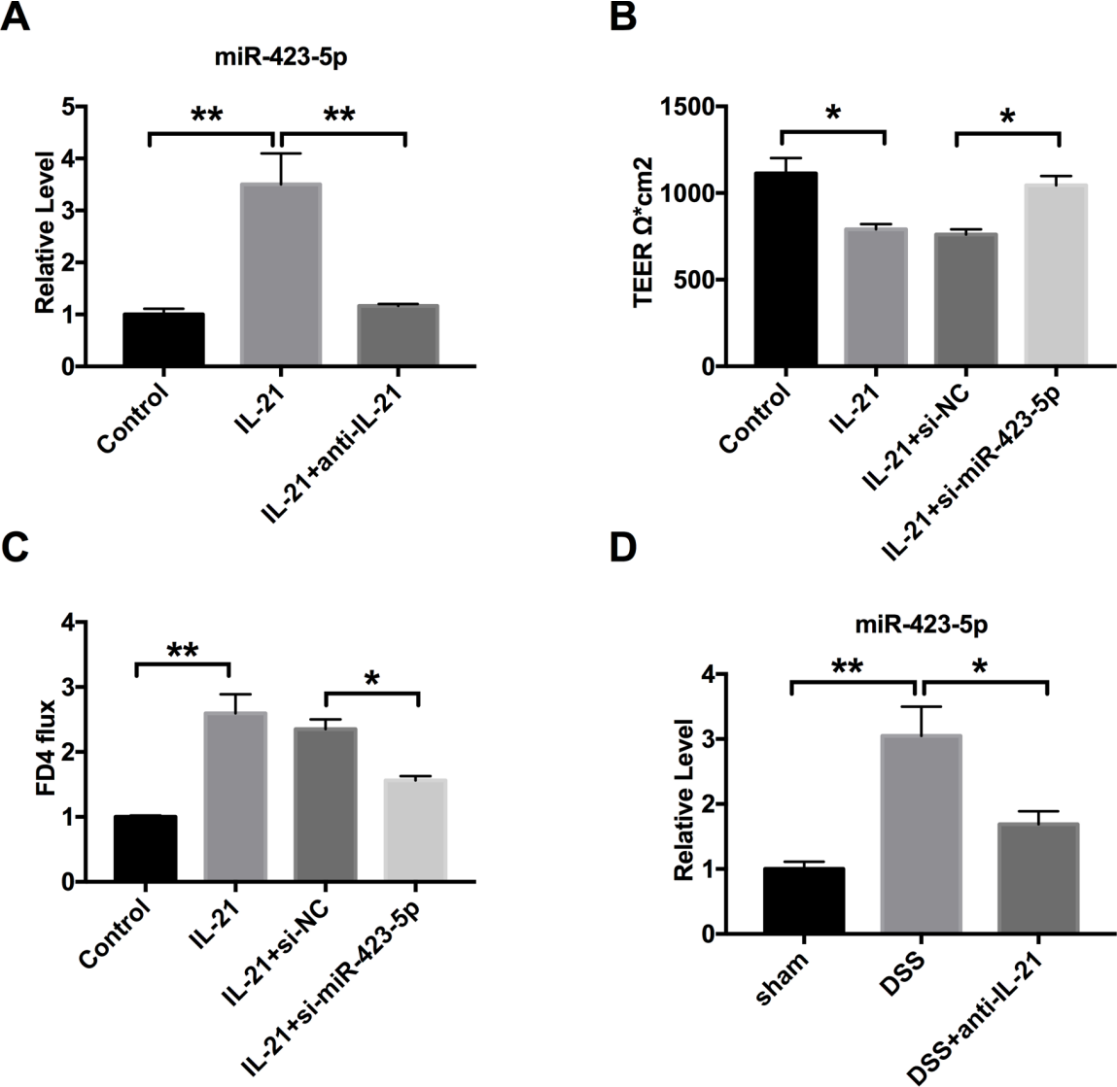
**IL-21 regulates miR-423-5p in UC.** (**A**) The expression of miR-423-5p in colorectal epithelial cell lines. n=8. (**B**) TEER was determined in Caco-2 cells. n=4. (**C**) The FD4 level was detected after si-miR-423-5p/si-NC transfection in IL-21 treated cells. n=5. (**D**) The expression of miR-423-5p in different groups. n=7. *P < 0.05, **P < 0.01.

Based on the above experimental results, we confirmed that miR-423-5p participated in UC progression. Next, we need to confirm the relationship between IL-21 and miR-423-5p. Colorectal epithelial cells were treated with IL-21 and IL-21 neutralizing antibody, the level of miR-423-5p was assessed by RT-PCR. We observed that IL-21 induced expression of miR-423-5p, and IL-21 neutralizing antibody blocked the expression of miR-423-5p (Figure 6A). Then the Caco-2 cells were transfection with si-miR-423-5p/si-NC after IL-21 treatment. By TEER detecting, we observed that si-miR-423-5p restored the injured-intestinal epithelial barrier function after IL-21 treatment (Figure 6B). Down-regulated of miR-423-5p recovered the change of FD4 level caused by IL-21 (Figure 6C). In vivo, compared with DSS mice, the level of miR-423-5p was down-regulated in DSS mice after IL-21 neutralizing antibody injection (Figure 6D). Taken together, miR-423-5p may be downstream of IL-21 and regulated by IL-21

## DISCUSSION

IL-21 triggers an inflammatory cascade leading to intestinal inflammation. We, for the first time, have identified miR-423-5p as a critical component of the IL-21 inflammatory cascade in IBD. MiR-423-5p functions as a pro-inflammatory microRNA and is tightly controlled by IL-21 in IBD. Pro-inflammatory miR-423-5p directly targets claudin-5 (CLDN5), a critical family member in the maintenance of normal intestinal barrier property, and regulated NF-κB/MAPKs/JNK signaling pathway. Our study characterizes this novel IL-21/miR-423-5p/CLDN5 pathway in the development of IBD.

IL-21 is a cytokine belonging to the IL-2 family cloned by Parrish-Novak et al. in CD3 T cells. Studies have found that there is a high expression of IL-21 in most patients with inflammatory bowel disease, indicating that IL-21 plays an essential role in the process of inflammatory bowel disease [11]. In other diseases mediated by immune system imbalance, such as rheumatoid arthritis, hepatitis B, and nephrotic syndrome, the amount of IL-21 in peripheral blood is also increased [12, 13]. T cell-mediated immune response plays a central role in the pathogenesis of IBD tissue destruction. It is not clear how T cells mediate tissue destruction in IBD, but there is evidence that T cell-derived cytokine IL-21 stimulates human fibroblasts to synthesize and secrete interstitial metalloproteins MMP-1, MMP-2, MMP-3 and MMP-9, which can directly degrade mucosa. The expression level of IL-21 in IBD is also increased, which may promote the destruction of digestive tract tissue. In addition, the study on the production of IL-21 protein in isolated monocytes of whole mucosa and lamina propria from autoimmune and inflammatory CD patients showed that IL-21 was highly expressed in the disease site compared with healthy controls, indicating that IL-21 promotes chronic progressive mucosal inflammation in CD [14, 15]. It has been proved that the active interaction between mucosal immune cells and non-immune cells promotes tissue destruction, and cytokines must be regulated. IL-21 is one of them, is overexpressed in CD tissues, and supports the response of Th1 cells to progressive inflammation. This suggests that IL-21 can regulate other inflammatory pathways in the digestive tract except to enhance the immune response of Th1 cells [16–18]. Similarly, with the previous study, we also found that IL-21 was upregulation in IBD mice. After treating with IL-21 neutralizing antibody, the mice showed a decreased level of inflammatory and in IBD mice.

Tight junction (TJ) exists between epithelial cells and between epithelial cells and vascular endothelial cells, which is responsible for regulating the transmembrane transport of ions and solute molecules and maintaining cell polarity and is related to extracellular and extracellular permeability [18]. Pathological factors such as hypoxia and physical and chemical injury affect tight junction function and structure. There are three kinds of junction complexes at the top of the lateral membrane of the adjacent cells between human epithelial cells, namely tight junction, adhesive junction and desmosome junction, which are mainly composed of three kinds of superfamily proteins, including transmembrane protein families: occludin and claudin and peri-membrane proteins (zonulae occludente, ZO) [19]. Studies have shown that the expression of claudin-2 in the colon of patients with active ulcerative colitis is significantly increased. Compared with UC, CD showed the main Th1 immune response. In this context, the central pro-inflammatory cytokines TNF α and interferon-γ have been shown to increase the expression of claudin-2 in HT-29/B6 and Caco-2 cells [20]. Decreased expression and localization of claudin-3 in the inflammatory colon of patients with CD. In summary, the claudin family plays a crucial role in IBD.

MicroRNA plays a vital role in many biological processes. Intestinal inflammation is an important aspect. Cellular stress, inflammation, and other factors can induce the expression of MicroRNA, thus affecting a variety of biological processes and play an inflammatory or anti-inflammatory role. The gene mutation and inactivation of MicroRNA will lead to down-regulation or overexpression of MicroRNA, which is closely related to intestinal immunity and inflammation. Sanctuary et al. [21] found that down-regulation of miR-106a expression can attenuate intestinal inflammation mediated by TNF- α, and miR-106a knockout can reduce chronic ileitis in mice, and out that it is related to the inhibition of post-transcriptional regulation of IL-10 release through the binding of NF- κB promoter, which proves that miR-106a has the potential to treat chronic inflammation including IBD. In addition, miR-16 is overexpressed in the colonic mucosa of patients with UC by inhibiting colonic mucosa. The expression of the A2a receptor inhibits the activation of the NF- κB signal pathway and regulates immune and inflammatory response [22]. In our model, however, we found that miR-423-5p is a pro-inflammatory miRNA. A similar pro-inflammatory role of miR-423-5p has been reported in other models [23]. Thus, future studies are needed to explore the miRNA/target RNA interactome network in the IL-21 pathway.

The intestinal tract is a relatively complex system in the human body, and it is the internal ecosystem of the human body, in which there is an ecological balance composed of countless kinds of bacteria. The barrier system of harmful substances in the intestinal tract of the human body is to protect human health. Important tools. In hemorrhagic shock. Sepsis, etc. [6]. It will cause damage to the intestinal tract of patients, especially for some elderly patients, because of their high age, low immunity, decreased body function, accompanied by the simultaneous existence of a variety of diseases, and the degradation of intestinal function, the intestinal barrier function is even more weakened. Therefore, it is of considerable significance for patients to judge early whether the patient has an intestinal mucosal injury and carry out effective intervention [24]. With the continuous in-depth study of intestinal barrier function in the clinic, unique and comprehensive testing can also be carried out. With the constant development of medicine, it provides a significant reference value for the clinical.

## MATERIALS AND METHODS

### Human colon samples

Human IBD colonic tissue was taken from IBD patients and healthy volunteers in Shanxi Provincial People's Hospital. All participants signed the informed consent form. The research scheme was approved by the Ethics Committee of Shanxi Provincial People's Hospital, and the experimental method was in line with the Helsinki Declaration.

### Cell cultures

Caco-2 cells were seeded in DMEM (Biological Industries, Israel) supplemented with 10% FBS (Biological Industries). SW480 cells were cultured in 1640 (Biological Industries, Israel) medium supplemented with 10% FBS. NCM460 cells were cultured in M3: BaseF medium (Biological Industries, Israel) supplemented with 10% FBS and 1% penicillin and streptomycin sulfate (Gibco, USA).

### Cell transfection

For the siRNA/mimic/plasmid transfection, 2 × 105 cells per well were plated in a 6-well plate. After adhering for 24 hours, si-miR-423-5p/si-NC, miR-NC/miR-423-5p, or claudin-5/vector (RiboBio, China) were added to the transfection medium with Lipofectamine 2000 (Thermo Fisher Scientific, USA) for 6 hours at 37°C in a CO2 incubator. After transfection, the cells were supplemented with a normal culture medium and cultured at 37°C/5% CO2 for up to 48 hours before harvest.

### Western blot

Total proteins were extracted from cells and tissues. The cells and tissues were lysed with 20 μL of RIPA Buffer (Roche Molecular Biochemicals, Switzerland), and protein concentrations were detected using the BCA kit (Beyotime, China), according to the manufacturer’s guidelines. Protein samples (20~40 μg) were separated on an SDS-PAGE gel and transferred to nitrocellulose membranes. After blocking the membranes with 5% skim milk in phosphate-buffered saline (PBS) for 1 h at room temperature, the membranes were probed with anti-IL-21 (Abcam, UK), anti-claudin-5 (GeneTex, USA), anti-p-NF-κB (Santa Cruz Biotechnology, USA), anti-NF-κB (Santa Cruz Biotechnology, USA), anti-p-ERK1/2 (Proteintech, USA), anti-ERK1/2 (Proteintech, USA), anti-p-JNK (Santa Cruz Biotechnology, USA), anti-JNK (Proteintech, USA), anti-p-P38 (Santa Cruz Biotechnology, USA), anti-P38 (GeneTex, USA) and GAPDH (Zhongshanjinqiao, China) antibodies at 4°C overnight. The nitrocellulose membranes were washed with PBST (PBS containing 0.5% Tween 20) three times for 7 min each, which was followed by the incubation with a fluorescence-labeled secondary antibody (IRDye700/800 mouse and rabbit antibodies) (Santa Cruz Biotechnology, USA). Protein levels were determined using an Odyssey infrared scanning system (LI-COR Biosciences, USA), and the band intensities were quantified using ImageJ software.

### Quantitative real-time PCR

Total RNA was isolated from cells and tissues using Trizol reagent (Invitrogen, USA) according to the manufacturer’s protocols. Total RNA (1 μg) was used for synthesizing the first-strand cDNA using the cDNA Reverse Transcription Kit (Applied Biosystems, USA). qRT-PCR was performed with the SYBR Green Mix kit (Applied Biosystems, USA) according to the manufacturer’s instructions. The relative RNA levels were calculated using the Δ ΔCt method. GAPDH levels served as an internal control. miR-423-5p expression was assessed using the qRT-PCR miRNA Detection Kit (Applied Biosystems, USA), with U6 levels as an internal control. Gene expression was calculated using the 2-ΔΔCt method. The primer sequences were shown as follow:

IL-21

Forward: 5'-TGTCGCTAGCTCCTGG AGACTCAGT TCTG-3';

Reverse: 5'-CCGGGATATCCTAGGAGAGATGCTG A TG-3';

claudin-1

Forward: 5'-ATCTCGAGATGGCCAACGCGGGGCTGCAG-3';

Reverse: 5'-GTGGATCCTCACACGTAGTCTTTCCCGCTG-3';

claudin-4

Forward: 5'-ACTGGTACCAGCATGGTTGGGAAGA GAGATG-3';

Reverse: 5'-ACGCTCGAGCAGTTTCTCTGGATTCC TGAG-3;

claudin-5

Forward: 5-GATTGGCTCTCGAGCGATGACCGGCG -3;

Reverse: 5-CGTGCCCAAGCTTCTCAGACGTAGTT C-3;

claudin-8

Forward: 5-TCTGCAGTAGGA- CATAGAAACCCC TAA-3;

Reverse: 5-CGTTTAGG GGTTTCTATGTCCTACTG C-3;

claudin-11

Forward: 5-CCGGTGTGGCTAAGTACA-3;

Reverse: 5-ACTGAACCTGACCGTACACACACAGG GAACCAGATG-3;

miR-423-5p

Forward:5-ATGGTTCGTGGGTGAGGGGCAGAGAGCGAGAGCAGGGTCCGAGGTATTCG-3;

Reverse: 5-GTGCAGGGTCCGAGGT-3;

### Dual Luciferase Reporter Assay

20 mmol/L miR-423-5p mimic/si- miR-423-5p or negative control (si-NC) and claudin-5 were co-transfected into HEK293T cells. Luciferase activity was detected with Luciferase Reporter Assay Kit (Biovision, China) on a luminometer (Berthold, Germany) 48 hr after the transfection.

### Detection of barrier permeability

For the establishment of a monolayer model of Caco-2 cells. The determination of transepithelial resistance of cells, also known as transepithelial electrical resistance (TEER), is currently recognized as a simple and authoritative method for evaluation. Its value can reflect the permeability of the cell monolayer. TEER is formed by the flow of ions through the paracellular space. Because of its simple operation and repeatability, it has become a common index for detecting cell bypass permeability. The transmembrane resistance of cells was measured by MillicelI-ERS transmembrane resistance meter. The transmembrane resistance of Caco-2 cells = (sample hole measured value-blank hole measured value) 2x Millicell membrane area = resistance value .cm2.

### Colitis model

Mice were purchased from Beijing Charles river and fed in the experimental Animal Center of Shanxi Provincial People's Hospital with the standard condition. Day 1 to Day 7: C57BL/6 in the model group was fed 3% Dextran Sodium Sulfate (DSS) aqueous solution, while the mice in the untreated group drank normal water. Day 8 to Day 14: replace fresh DSS-free drinking water. On day 14, mice were intraperitoneally injected with 3% pentobarbital sodium and were killed by excessive anesthesia with a dose of 90 mL/kg, and the organ and tissue were removed for follow-up study. The research protocol of this study was approved by the Animal Care and Use Committee of Shanxi Provincial People's Hospital.

Mice were treated with IL-21-neutralizing antibodies (0.5 mg/kg) by intraperitoneal injection at day 8-10 after DSS treated, at day 14, mice were intraperitoneally injected with 3% pentobarbital sodium and were killed by excessive anesthesia with a dose of 90 mL/kg, and the organ and tissue were removed for follow-up study.

For inhibition of miR-423-5p, mice in the antagomir-423-5p (50 nmol, RiBoBio, China) group were dosed by tail vein injection on the 7^th^ day.

### Measurement of myeloperoxidase activity

Myeloperoxidase (MPO) activity was measured in the distal colonic tissue obtained from control and mice with colitis. The inflamed distal colon (5 cm) was removed. MPO is an enzyme found primarily in neutrophils; measurement of MPO has been widely used as a marker for intestinal inflammation [25]. MPO activity was measured according to the protocol described by Krawisz et al. [26]. Briefly, after the samples were weighed, tissue samples were homogenized in a buffer (0.5% hexadecyltrimethylammonium bromide in 50 mM potassium phosphate buffer, pH 6.0) for 1 min. The samples were frozen in liquid nitrogen, thawed three times, and centrifuged at 20,000 × g for 20 min at 4°C using a microcentrifuge. Aliquots of supernatants (20 ml) were mixed with 980 ml of O-dianisidine. Absorbance was recorded at 450 nm every 1 min over a period of 10 min by ELISA. MPO activity was expressed as units/g of tissue. An enzyme unit was defined as the conversion of 1 mol of H2O2 per min at 25°C.

### Statistical analysis

Data were calculated as means ± SEM and analyzed by GraphPad 7.0. T-test and two-way ANOVA were used to analyze the data P < 0.05 was considered as a statistically significant difference.
